# FOXP4-AS1 May be a Potential Prognostic Biomarker in Human Cancers: A Meta−Analysis and Bioinformatics Analysis

**DOI:** 10.3389/fonc.2022.799265

**Published:** 2022-05-26

**Authors:** Guangming Zhang, Yongfeng Wang, Xiaoyong Han, Tingting Lu, Liangyin Fu, Haojie Jin, Kehu Yang, Hui Cai

**Affiliations:** ^1^ The First Clinical Medical College of Gansu University of Chinese Medicine (Gansu Provincial Hospital), Lanzhou, China; ^2^ Department of General Surgery Clinical Medical Center, Gansu Provincial Hospital, Lanzhou, China; ^3^ Key Laboratory of Molecular Diagnostics and Precision Medicine for Surgical Oncology in Gansu Province, Lanzhou, China; ^4^ Evidence-Based Medicine Center, School of Basic Medical Sciences, Lanzhou University, Lanzhou, China; ^5^ Ning Xia Medical University, Yinchuan, China; ^6^ Institution of Clinical Research and Evidence Based Medicine, Gansu Provincial Hospital, Lanzhou, China; ^7^ The First Clinical Medical College of Lanzhou University, Lanzhou, China; ^8^ Key Laboratory of Evidence Based Medicine and Knowledge Translation of Gansu Province, Lanzhou, China

**Keywords:** lncRNA, FOXP4-AS1, cancers, prognosis, meta-analysis, bioinformatics analysis

## Abstract

**Background:**

Cancer is one of the leading causes of death worldwide. Early diagnosis can significantly lower cancer-related mortality. Studies have shown that the lncRNA Forkhead box P4 antisense RNA 1 (FOXP4-AS1) is aberrantly expressed in various solid tumors. A meta-analysis was performed to evaluate the correlation of FOXP4-AS1 with the prognosis of cancer patients and determine the clinical value of FOXP4-AS1 as a potential diagnostic marker.

**Methods:**

Correlational studies from the Web of Science, Embase, OVID, Cochrane and PubMed databases were screened (up to April 1, 2021). Meta-analysis was performed using Stata SE12.0 software.

**Results:**

Eleven original studies with 1,332 patients who were diagnosed with a solid cancer (nasopharyngeal carcinoma, hepatocellular carcinoma, colorectal cancer, gastric cancer, osteosarcoma, mantle cell lymphoma, prostate cancer, and pancreatic ductal adenocarcinoma) were included in the meta-analysis. High expression of FOXP4-AS1 was correlated with poor overall survival (OS) (HR = 1.77, 95% CI 1.29–2.44, *P* < 0.001) and shorter disease−free survival (DFS) (HR = 1.66, 95% CI 1.01–2.72, *P* = 0.044). Subgroup analysis based on sample size, follow-up time and Newcastle-Ottawa Scale (NOS) score revealed significant differences between FOXP4-AS1 levels and OS (*P* < 0.05). However, the expression level of FOXP4-AS1 was not significantly correlated with the OS of gastric cancer patients (*P* = 0.381). High expression of FOXP4-AS1 was predictive of a larger tumor size (OR = 3.82, 95% CI 2.3–6.3, *P* < 0.001).

**Conclusions:**

Overexpression of FOXP4-AS1 correlates with poor prognosis of cancer patients, and is a potential prognostic biomarker and therapeutic target.

**Systematic Review Registration:**

PROSPERO, identifier CRD42021245267.

## Introduction

Cancer is one of the leading causes of death worldwide. An estimated 19.3 million new cases of cancer were diagnosed in 2020 alone, and 10.0 million cancer-related deaths were recorded the same year. Furthermore, the global incidence and mortality rates of cancer are steadily increasing (1). Around 4.6 million newly diagnosed cases and 3.0 million cancer-related deaths were recorded in China in 2020 (2). Despite improvements in treatment strategies such as surgery, chemoradiotherapy and targeted therapy, the long‐term survival rate of cancer patients is still very low due to malignant progression of tumor cells and the lack of effective early diagnosis (3, 4). Thus, it is essential to identify new biomarkers and therapeutic targets for monitoring cancer development and improving clinical outcomes.

LncRNAs are single-stranded non-coding RNAs that are longer than 200 nucleotides and lack open reading frame. LncRNAs are involved in the regulation of many cellular processes, such as cell differentiation and development, and immune response (5, 6). Numerous studies have shown that lncRNAs can be crucial players in the occurrence and development of human tumors by acting as oncogenes or cancer suppressor genes (7). Moreover, lncRNAs can be considered useful markers for cancer diagnosis and prognosis (8–10).

The lncRNA FOXP4-AS1 modulates various physiological activities such as proliferation, invasion, migration and apoptosis (11). Li et al. reported that FOXP4-AS1 was overexpressed in colorectal cancer (CRC) tissues and closely associated with cell cycle progression (12). Recent studies have verified the overexpression of FOXP4-AS1 in multiple cancers, such as osteosarcoma (OSA) (13), prostate cancer (PCa) (14), nasopharyngeal carcinoma (NPC) (15, 16), hepatocellular carcinoma (HCC) (17, 18), pancreatic ductal adenocarcinoma (PDAC) (19), gastric cancer (GC) (20, 21), cervical cancer (CC) (11), mantle cell lymphoma (MCL) (22). Moreover, FOXP4-AS1 overexpression in cancer patients displayed poor prognosis. However, these studies were based on small patient samples and therefore inconclusive. The aim of this meta-analysis was to determine the prognostic value of FOXP4-AS1 in cancer.

## Materials and Methods

### Registration

The study was registered on PROSPERO (The registration number is: CRD42021245267).

### Search Strategy

By using Web of Science, Embase, OVID, Cochrane, and PubMed, we conducted a complete and thorough literature search (up to April 1, 2021). Our search keywords are as follows: (“FOXP4-AS1” OR “long noncoding RNA FOXP4-AS1” OR “lncRNA FOXP4-AS1” OR “forkhead box P4 antisense RNA 1”) AND (“neoplasm” OR “tumor” OR “malignancy” OR “neoplasia” OR “melanoma” OR “cancer” OR “sarcoma” OR “carcinoma” OR “adenoma”). In addition, the references and review articles were manually screened to find other related articles (23, 24).

### Inclusion and Exclusion Criteria

Inclusion criteria for this study were as follows: (a) assessment of the association of FOXP4-AS1 expression with cancer, (b) stratification of patients into low and high FOXP4-AS1 expression groups, (c) description of prognostic indicators or relevant clinicopathologic parameters, and (d) adequate data for the calculation of odds ratio (OR) or hazard ratio (HR) with 95% confidence interval (CI).

Exclusion criteria were as follows: (a) case reports, conference abstracts, reviews, editorials, and non-human studies; (b) duplicate publications; (c) studies lacking clinical data; (d) non-English articles.

### Date Extraction and Quality Assessment

Two researchers independently extracted the data from each study (25, 26), and any disagreement was resolved by discussing with a third author (27). We fetched the following information and data from each study: (a) the first author’s last name, (b) publication year, (c) country of publication, (d) cancer type, (e) sample type, (f) number of samples, (g) FOXP4-AS1 expression detection technique, (h) cut-off value, (i) follow-up times, and (j) clinicopathological features. Data regarding overall survival (OS), progression-free survival (PFS) or disease−free survival (DFS) data were directly obtained, or extracted from the Kaplan-Meier (K-M) curves using Engauge Digitizer software, and the HRs and 95% CIs were computed. The quality of each study was independently evaluated by two reviewers utilizing the Newcastle-Ottawa Scale (NOS). The total NOS score is 0 ~ 9, with ≥ 7 were considered of high quality.

### Statistical Methods

Stata SE12.0 was used for all statistical analyses. OS, DFS and PFS were analyzed using HR and clinicopathological parameters using OR. The chi-squared test and *I^2^* statistics were used for analyzing heterogeneity (28). The random-effect model was applied for variables with strong heterogeneity (chi-squared test, *P_Q_* < 0.1, *I^2^* > 50%), and the fixed‐effect model was used otherwise. Data were presented in forest plots. The Egger’s test and Begg’s funnel plot were employed to evaluate publication bias, and sensitivity analysis was conducted to assess the robustness of the findings. *P* < 0.05 was defined as statistically significant.

### Target Gene Prediction and Signal Pathway Network Construction

Related genes for FOXP4-AS1 were downloaded from the MEM-Multi Experiment Matrix database. Next, Gene Ontology (GO) and the Kyoto Encyclopedia of Genes and Genomes (KEGG) pathway enrichment analysis were conducted by using R software (*P* < 0.05). Moreover, a signaling pathway network was created utilizing Cytoscape software.

## Results

### Characteristics of Studies

**Figure 1:**
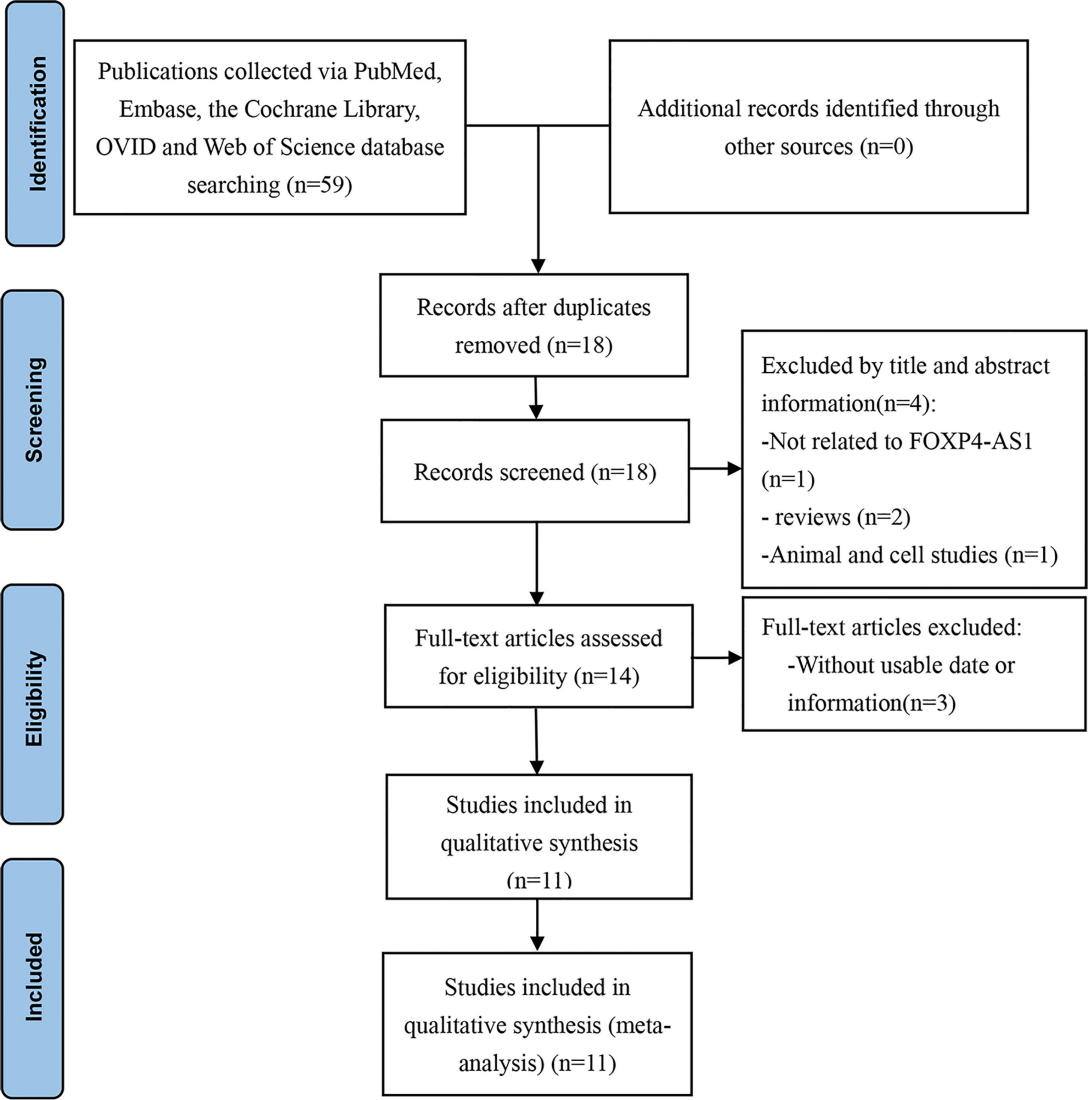
Flow diagram of this meta-analysis.

As shown in **Figure 1**, the initial search resulted in 59 articles, of which 41 were excluded as they were duplicate studies. Another four articles were eliminated after screening the title and abstracts because of irrelevant findings and non-human study subjects. The remaining 14 articles were evaluated in detail, and three were excluded due to missing data or information. Finally, 11 articles (12–22) were used for the current meta-analysis.

The studies included 1,332 patients and were published between 2017 and 2021. All studies were performed in China. The sample size of these studies varied from 24 to 384. Among the eleven studies, one focused on CRC (12), one on OSA (13), one on PCa (14), two on NPC (15, 16), two on HCC (17, 18), one on PDAC (19), two on GC (20, 21), and one on MCL (22). FOXP4-AS1 expression was measured using real-time quantitative polymerase chain reaction (RT-qPCR). The characteristics of the included articles are summarized in **Table 1**.

**Table 1:** Characteristics of studies in this meta-analysis.

Study	Year	Country	Cancertype	Sample type	Total Size(n)	Detectionmethod	Cutoff	Outcome	Multivariateanalysis	HRstatistic	NOS score
Binang (20)	2020	China	GC	tissue	61/384	RT-qPCR	NR	OS/DFS	No	NR	7
Chen (21)	2019	China	GC	tissue	24	RT-qPCR	NR	OS	No	SC	6
Li (12)	2017	China	CRC	tissue	48	RT-qPCR	median	NR	No	NR	6
Liang (17)	2021	China	HCC	tissue	121	RT-qPCR	NR	OS/DFS	Yes	Rep	8
Wang (18)	2019	China	HCC	tissue	213	RT-qPCR	mean	OS/DFS	Yes	Rep	7
Wu (14)	2019	China	PCa	tissue	64	RT-qPCR	median	OS	No	SC	7
Yao (15)	2021	China	NPC	blood	166	RT-qPCR	median	OS/PFS	Yes	Rep	8
Zhong (16)	2020	China	NPC	tissue	80	RT-qPCR	median	OS	Yes	SC	7
Yang (13)	2018	China	OSA	tissue	60	RT-qPCR	NR	OS/DFS	Yes	SC	6
Tao (22)	2021	China	MCL	blood	60	RT-qPCR	median	OS/DFS	Yes	Rep	6
Liu (19)	2019	China	PDAC	tissue	112	NR	median	OS	Yes	Rep	7

HR, hazard ratio; GC, gastric cancer; CRC, colorectal cancer; HCC, hepatocellular carcinoma; PCa, prostate cancer; NPC, nasopharyngeal carcinoma; OSA, osteosarcoma; MCL, mantle cell lymphoma; PDAC, pancreatic ductal adenocarcinoma; NR, no report; OS, overall survival; DFS, disease−free survival; PFS, progression-free survival; Rep, report; SC, survival curve; RT-qPCR, real-time quantitative polymerase chain reaction.

### Association Between FOXP4-AS1 Expression and Overall Survival

**Figure 2:**
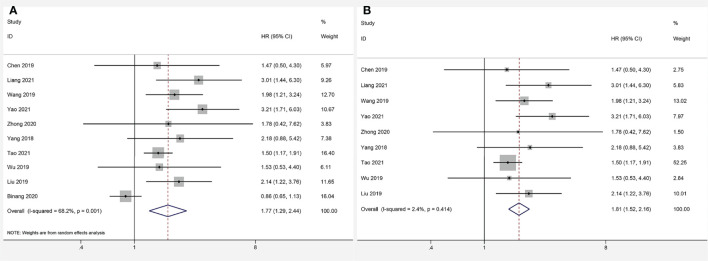
Forest plots for association of FOXP4-AS1 expression with overall survival. **(A)** Forest plots of the entire literature. **(B)** Forest plots after exclusion of Binang et al, 2020.

**Figure 3:**
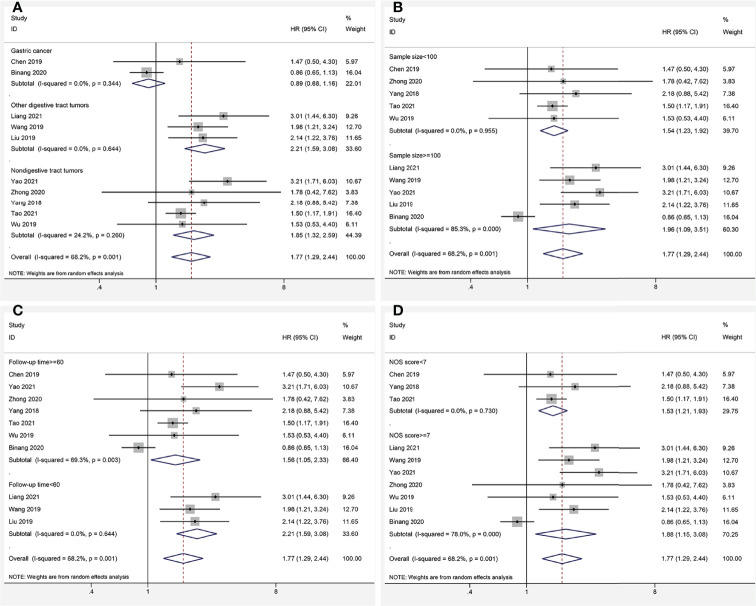
Forest plots for subgroup analysis of FOXP4-AS1 expression with overall survival. **(A)** Subgroup analysis stratified by cancer type. **(B)** Subgroup analysis stratified by sample size. **(C)** Subgroup analysis stratified by follow-up time. **(D)** Subgroup analysis stratified by NOS score.

Data from ten articles (13–22) including 1,284 cancer patients were assessed for the correlation between expression level of FOXP4-AS1 and OS. The random-effects model was employed because of the high heterogeneity (*I^2^* = 68.2%, *P_Q_* = 0.001). Overexpression of FOXP4-AS1 was strongly interrelated with worse OS (HR = 1.77, 95% CI 1.29–2.44, *P* < 0.001) (**Figure 2A**). The potential sources of heterogeneity were determined *via* sensitivity analysis. Omitting the study by Binang et al. (20) eliminated the heterogeneity (*I^2^* = 2.4%, *P_Q_* = 0.414) without affecting the results (HR = 1.81, 95% CI 1.52–2.61, *P* < 0.001) (**Figure 2B**). Subgroup analysis based on the tumor type (gastric cancer, other digestive tract tumors and nondigestive tract tumors), sample size (n ≥ 100 or n < 100), follow up times (≥ 60 months or < 60 months) and the NOS score (NOS scores ≥ 7 or < 7) further confirmed that FOXP4-AS1 overexpression correlated with poor OS of cancer patients in all subgroups except for gastric cancer (HR = 0.89, 95% CI 0.68–1.16, *P* = 0.381) (**Figure 3**). In addition, the heterogeneity was significantly reduced when analyzing cancer subgroups (**Table 2**). We then performed a meta-regression using the above-mentioned covariates but failed to identify the source of obvious heterogeneity (**Table 2**).

**Table 2:** Subgroup meta-analysis of pooled HRs for OS.

Stratified analysis	Studies (n)	Number of patients	Pooled HR (95% CI)	P-value	Meta regression (p value)	Heterogeneity	
						*I^2^* (%)	P-value
Cancer type					0.154		
Gastric cancer	2	408	0.89(0.68–1.16)	0.381	—	0	0.344
Other digestive tract tumors	3	446	2.21(1.59–3.08)	<0.001	—	0	0.644
Nondigestive tract tumors	5	430	1.85(1.32–2.59)	<0.001	—	24.2	0.260
Sample size					0.695		
≥100	5	996	1.96(1.09-3.51)	0.024	—	85.3	0.000
<100	5	288	1.54(1.23-1.92)	<0.001	—	0.0	0.955
Follow-up time					0.256		
≥60	7	224	1.56(1.05–2.33)	0.028	—	69.3	0.003
<60	3	564	2.21(1.59–3.08)	<0.001	—	0.0	0.644
NOS score					0.761		
≥7	7	838	1.88(1.15–3.08)	0.012	—	78.0	0.001
<7	3	446	1.53(1.21–1.93)	<0.001	—	0.0	0.730

### Association Between FOXP4-AS1 and Disease-Free Survival

**Figure 4:**
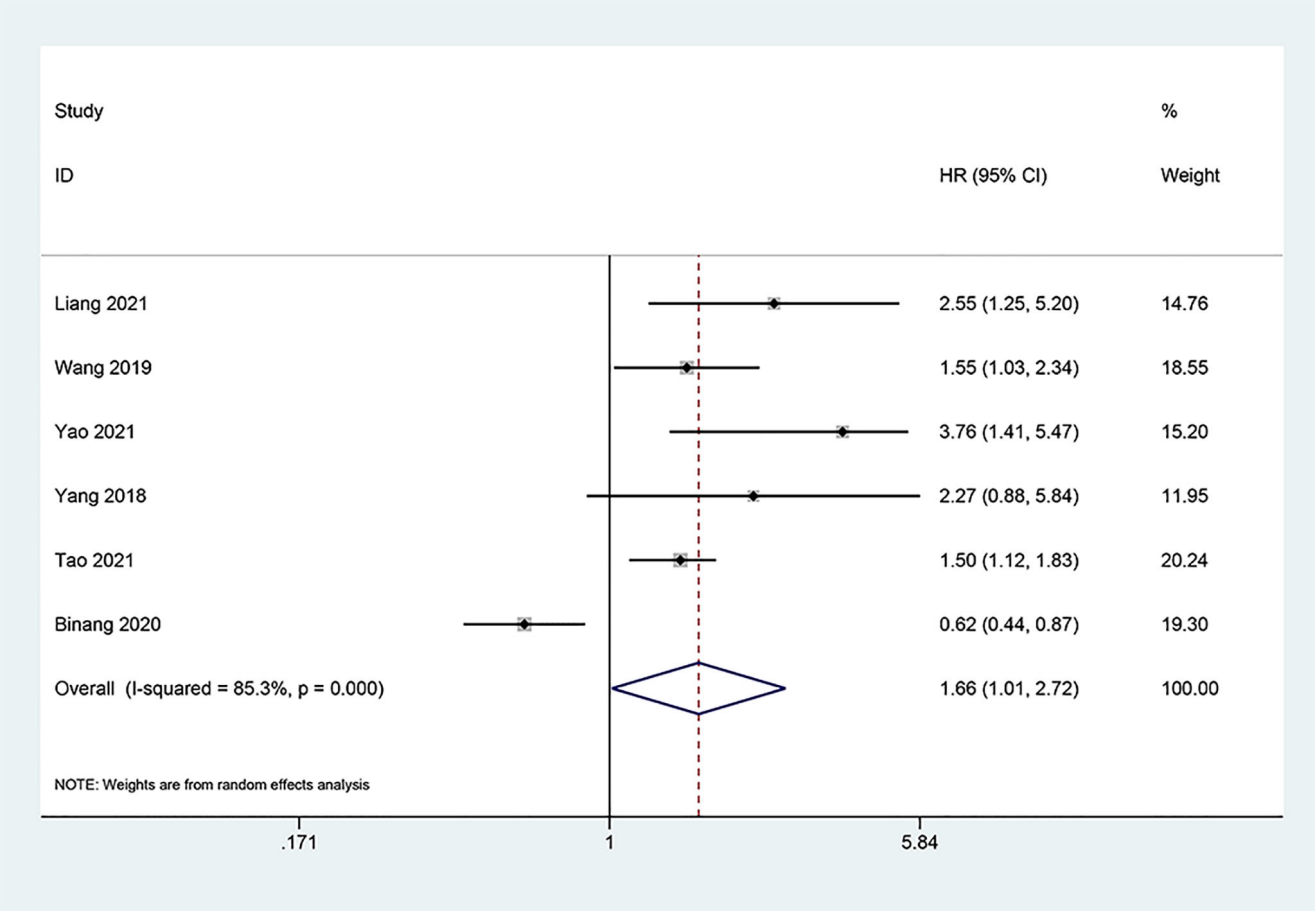
Forest plots for association of FOXP4-AS1 expression with disease-free survival.

Six studies (13, 15, 17, 18, 20, 22) consisting of 1,004 cancer patients analyzed the correlation of FOXP4-AS1 overexpression with DFS. Due to significant heterogeneity (*I^2^* = 85.3%, *P_Q_* < 0.001), the HR was pooled employing the random-effect model, and indicated that overexpression of FOXP4-AS1 was significantly correlated with shorter DFS (HR = 1.66, 95% CI 1.01–2.72, *P* = 0.044) (**Figure 4**).

### Association Between FOXP4-AS1 Expression Levels With OS of Certain Types of Cancers

**Figure 5:**
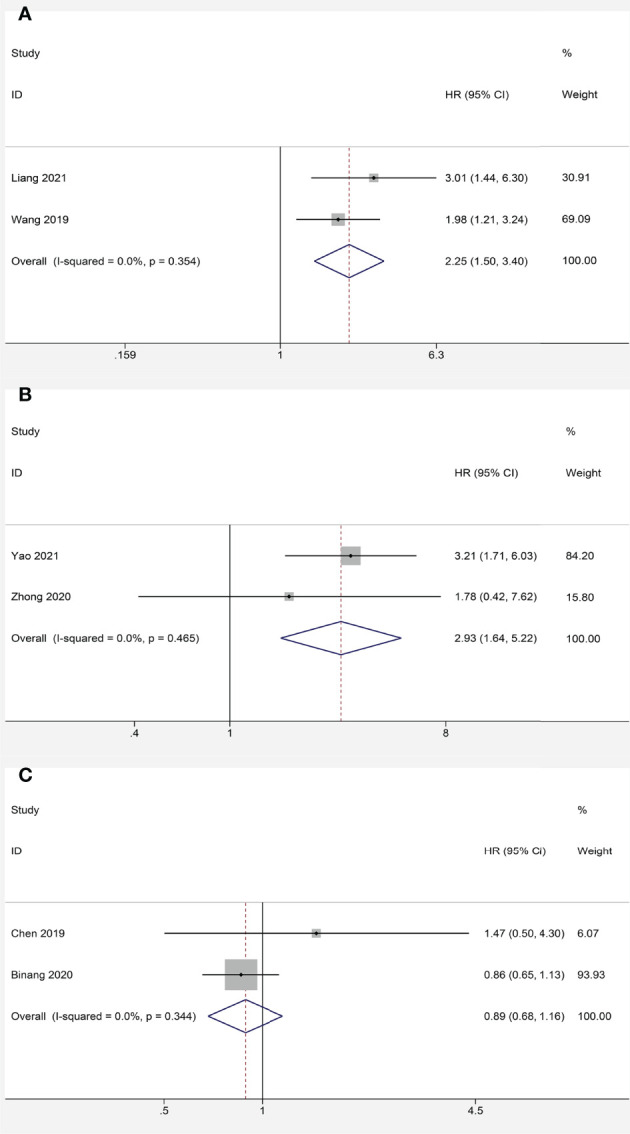
Meta-analysis of the pooled HR of OS for hepatocellular carcinoma **(A)** nasopharyngeal carcinoma **(B)** and gastric cancer **(C)**.

We further identified the prognostic value of FOXP4-AS1 in different cancers. FOXP4-AS1 overexpression was interrelated with lower OS in HCC (HR = 2.25, 95% CI 1.50–3.40, *P* < 0.001) (**Figure 5A**) and NPC (HR = 2.93, 95% CI 1.64–5.22, *P* < 0.001) (**Figure 5B**). In contrast, no significant correlation was observed in FOXP4-AS1 expression and the OS of GC patients (HR = 0.89, 95% CI 0.68–1.16, *P* = 0.381) (**Figure 5C**).

### Association Between FOXP4-AS1 and Clinicopathological Features

**Figure 6:**
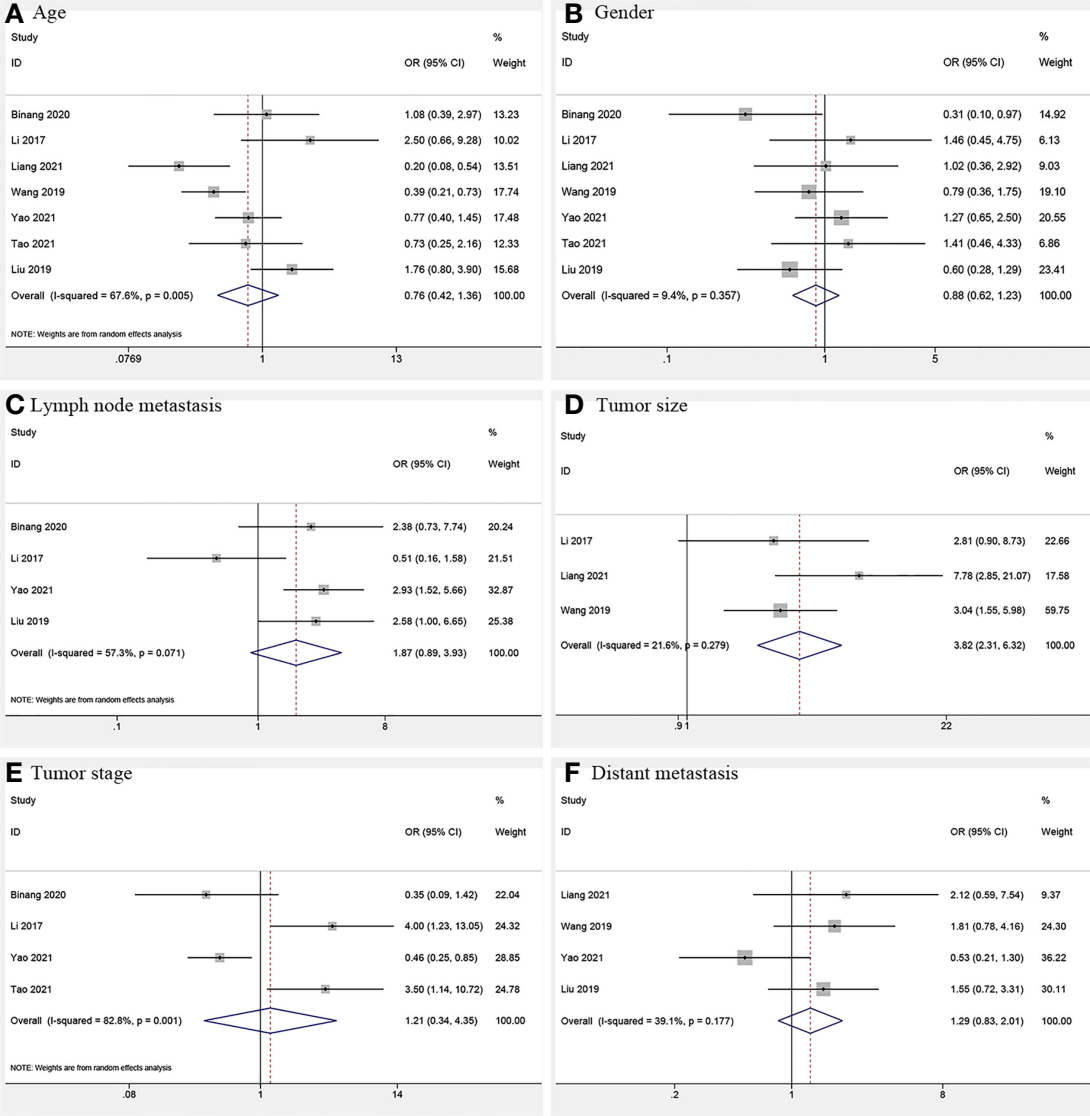
Forest plots for association of FOXP4-AS1 expression with clinicopathological features: **(A)** Age. **(B)** Gender. **(C)** Lymph node metastasis. **(D)** Tumor size. **(E)** Tumor stage. **(F)** Distant metastasis.

Three studies (12, 17, 18) consisting of 382 cancer patients analyzed the correlation of FOXP4-AS1 expression with tumor size. In the fixed-effects model (*I^2^* = 21.6%, *P_Q_* = 0.279), FOXP4-AS1 overexpression was interrelated with larger tumors (≥ 5 cm; OR = 3.82, 95% CI 2.31–6.32, *P* < 0.001) (**Figure 6**). We also investigated the correlation of FOXP4-AS1 expression with age (OR = 0.76, 95% CI 0.42–1.36, *P* = 0.356), gender (OR = 0.88, 95% CI 0.63–1.23, *P* = 0.451), lymph node metastasis (LNM) (OR = 1.87, 95% CI 0.89–3.93, *P* = 0.100), tumor stage (OR = 1.21, 95% CI 0.34–4.35, *P* = 0.768) and distant metastasis (OR = 1.29, 95% CI 0.83–2.02, *P* = 0.254) using ORs and the 95% CIs, but no significant correlation was observed (**Figure 6**).

### Risk of Bias and Sensitivity Analysis

**Figure 7:**
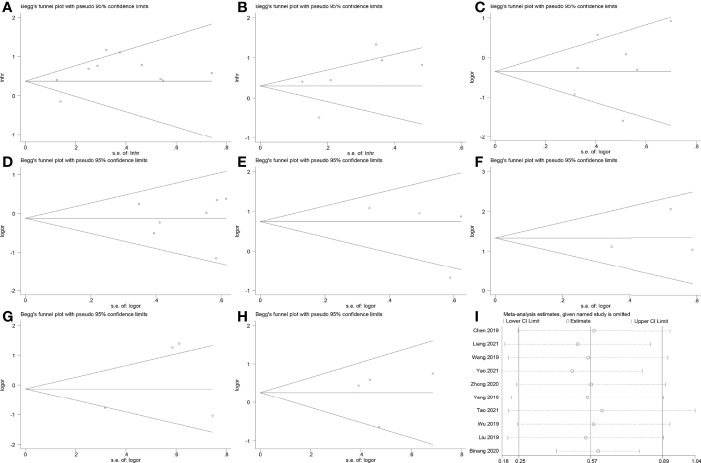
Begg’s funnel plots and sensitivity analysis. **(A)** Begg’s funnel plot for OS. **(B)** Begg’s funnel plot for DFS. **(C)** Begg’s funnel plot for age. **(D)** Begg’s funnel plot for gender. **(E)** Begg’s funnel plot for lymph node metastasis. **(F)** Begg’s funnel plot for tumor size. **(G)** Begg’s funnel plot for tumor stage. **(H)** Begg’s funnel plot for distant metastasis. **(I)** Sensitivity analysis for studies about OS.

The Egger’s test and Begg’s funnel plot did not reveal evident publication bias for OS (Pr > |t| = 0.101), DFS (Pr > |t| = 0.352), age (Pr > |t| = 0.491), gender (Pr > |t| = 0.885), LNM (Pr > |t| = 0.395), tumor size (Pr > |t| = 0.735), tumor stage (Pr > |t| = 0.440) and distant metastasis (Pr > |t| = 0.928) (**Figure 7A–H**). Sensitivity analysis of OS was conducted by the leave-one-out method to assess the effect of an individual article on the summarized results. However, no single article significantly altered the results (**Figure 7I**).

### Analysis of FOXP4-AS1-Related Genes

**Figure 8:**
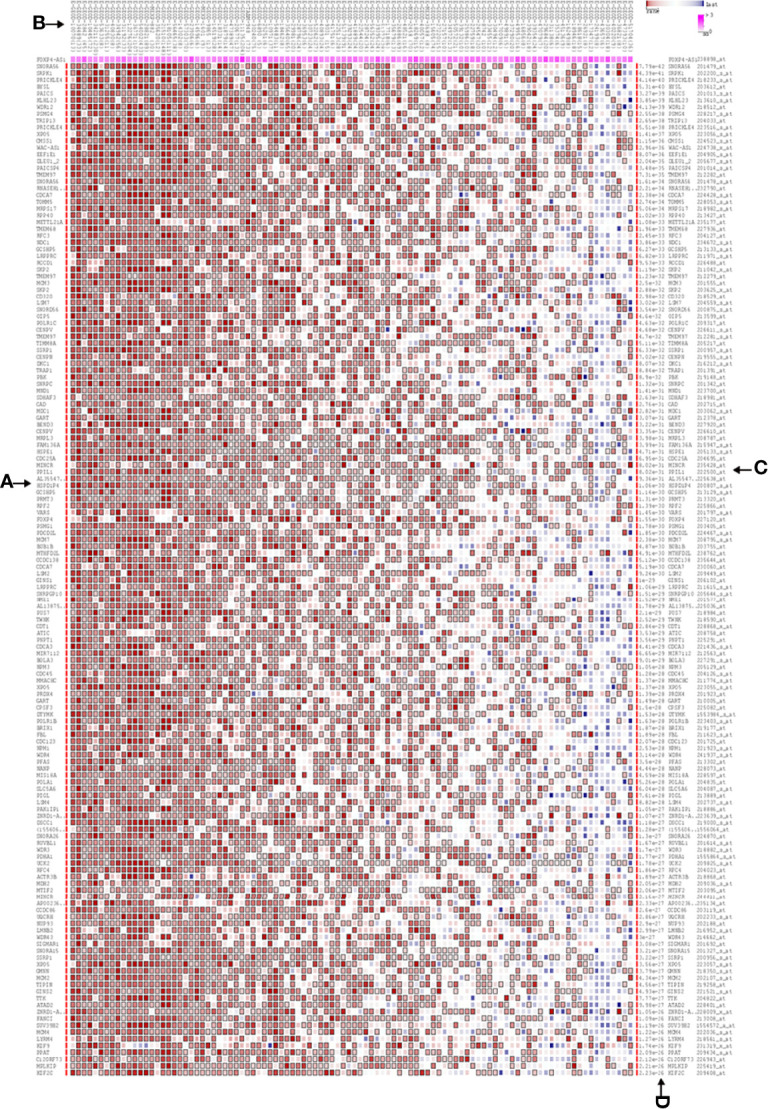
The top 150 predicted target genes of FOXP4-AS1 by using Multi Experiment Matrix (MEM, http://biit.cs.ut.ee/mem/) website. **(A)** predicted target genes; **(B)** Single experimental data set; **(C)** Gene probes; **(D)** P values.

**Figure 9:**
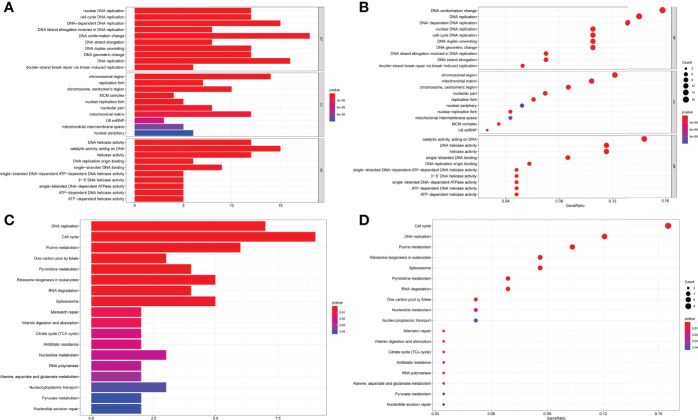
GO terms and the KEGG pathway. **(A)** Histogram presentation of the top 10 positions of GO terms of target genes in biological processes (BP), cellular components (CC) and molecular functions (MF) of biology; **(B)** Bubble chart of the top 10 positions of GO terms of target genes in BP, CC, MF; **(C)** Histogram presentation of pathways related to the differentially expressed genes by the KEGG analysis; **(D)** Bubble chart of pathways related to the differentially expressed genes by the KEGG analysis.

**Figure 10:**
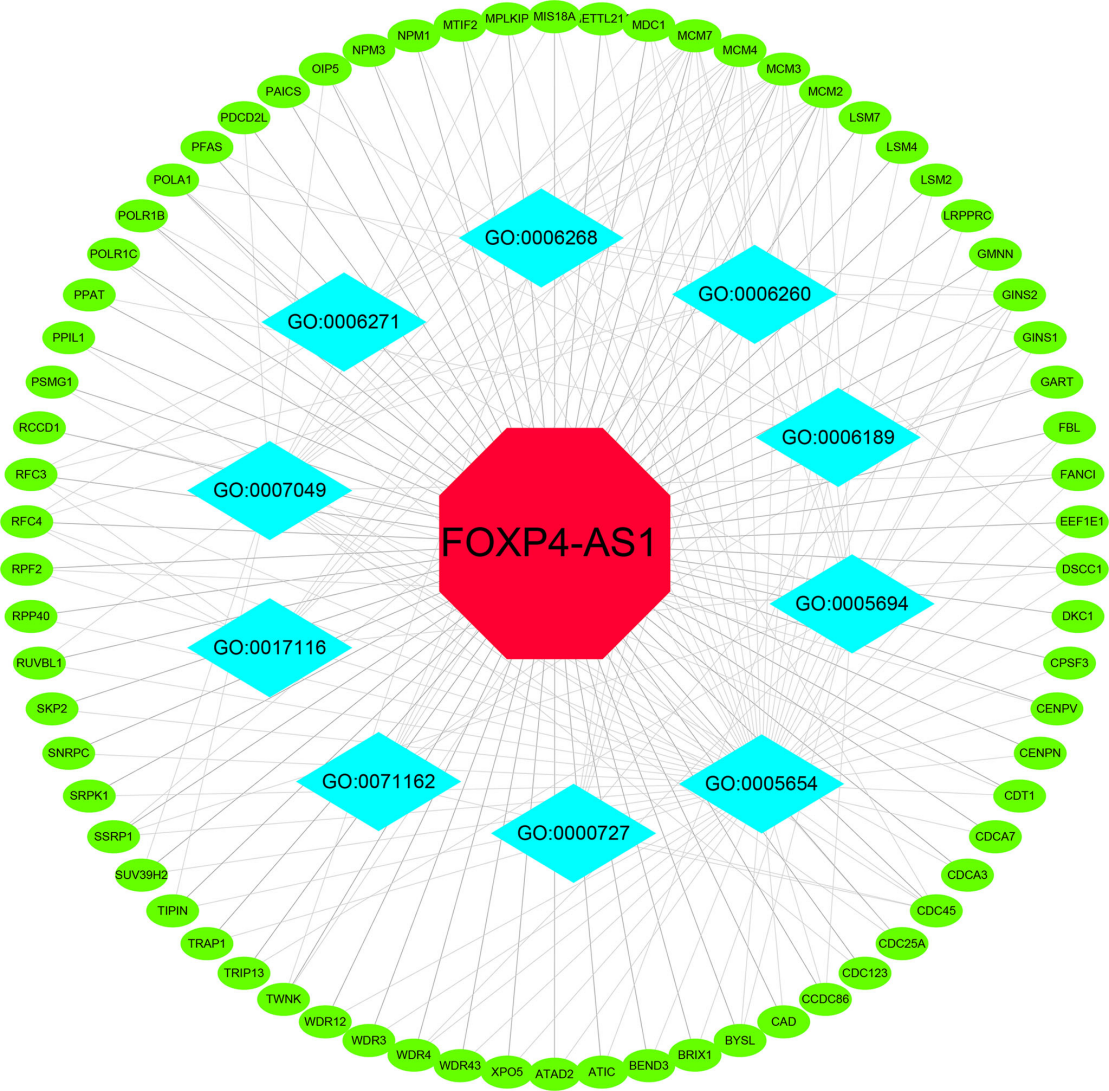
Differentially expressed gene interaction network analysis. Green nodes represent target genes and sky blue nodes represent the related pathway. GO:0005654 (nucleoplasm); GO:0005694 (chromosome); GO:0071162 (CMG complex); GO:0007049 (cell cycle); GO:0006268 (DNA unwinding); GO:0006271 (DNA strand elongation); GO:0017116 (ATP-dependent DNA helicase activity); GO:0000727 (double-strand break repair); GO:0006189 (‘*de novo*’ IMP biosynthetic process); GO:0006260 (DNA replication).

The MEM database was used to screen the top 150 co-expressed genes of FOXP4-AS1. *SNORA56*, *SRPK1* and *PRICKLE4* were the top three target genes ranked by p-value, and markedly associated with FOXP4-AS1 gene expression (**Figure 8**). To explore the underlying molecular mechanisms, GO and KEGG pathway analyses were performed. The results of GO analysis showed that co-expressed genes were mainly involved in biological processes (BP), such as nuclear DNA replication, DNA conformation change, and DNA duplex unwinding; cellular component (CC), such as chromosomal region, chromosomal region, and mitochondrial matrix; molecular function (MF), such as single−stranded DNA binding, DNA replication origin binding, and DNA helicase activity (**Figure 9**). Moreover, the KEGG pathway analysis results showed that co-expressed genes were principally implicated in DNA replication, cell cycle, and mismatch repair (**Figure 9**). Additionally, a signal pathway network was created using Cytoscape software (**Figure 10**; **Table 3**).

**Table 3:** Gene ontology analysis of FOXP4-AS1-related genes.

GO number	Description	Genes	P Value	FDR
**GO:0005654**	nucleoplasm	MDC1, SUV39H2, MCM7, WDR3, DSCC1, WDR4, GMNN, WDR43, FBL, RUVBL1, XPO5, OIP5, SKP2, TIPIN, RFC3, RFC4, CAD, CDC25A, DKC1, POLR1B, POLR1C, MCM3, MCM4, CCDC86, EEF1E1, SNRPC, MCM2, CDCA7, MTIF2, METTL21A, RPF2, CDC45, BEND3, FANCI, GINS1, TRAP1, GINS2, CDT1, NPM1, PPIL1, CENPV, CPSF3, RPP40, ATAD2, SSRP1, WDR12, NPM3, LSM4, LRPPRC, BYSL, SRPK1, LSM2, POLA1, LSM7, MIS18A, MPLKIP, CENPN, PSMG1	4.01E-13	6.33E-11
**GO:0005694**	chromosome	GINS2, MDC1, MCM7, WDR4, SSRP1, RPF2, BYSL, FBL, POLR1B, MCM3, CCDC86, MCM4, BRIX1, TRIP13, RCCD1, MCM2	1.27E-11	7.97E-10
**GO:0071162**	CMG complex	GINS1, GINS2, CDC45, MCM7, MCM3, MCM4, MCM2	1.51E-11	7.97E-10
**GO:0007049**	cell cycle	FANCI, SUV39H2, CDT1, MDC1, CDC123, CENPV, TIPIN, CDCA3, MCM7, CDC25A, PDCD2L, MIS18A, CDC45, RUVBL1, MCM3, MPLKIP, MCM4, OIP5, MCM2	2.01E-12	9.85E-10
**GO:0006268**	DNA unwinding	GINS1, GINS2, CDC45, MCM7, TWNK, MCM3, MCM4, MCM2	3.85E-11	9.43E-09
**GO:0006271**	DNA strand elongation	POLA1, RFC3, RFC4, MCM7, MCM3, MCM4	7.05E-10	1.15E-07
**GO:0017116**	ATP-dependent DNA helicase activity	RFC3, RFC4, MCM7, DSCC1, MCM3, MCM4, MCM2	1.69E-09	3.43E-07
**GO:0000727**	double-strand break repair	GINS2, CDC45, MCM7, MCM3, MCM4, MCM2	4.37E-09	5.34E-07
**GO:0006189**	‘*de novo*’ IMP biosynthetic process	ATIC, PPAT, GART, PFAS, PAICS	1.53E-08	1.5E-06
**GO:0006260**	DNA replication	GINS2, POLA1, RFC3, RFC4, MCM7, DSCC1, TWNK, MCM4, SSRP1, MCM2	2.78E-08	2.27E-06

### Molecular Mechanisms of FOXP4-AS1 Carcinogenesis in Various Cancers

Upregulation of FOXP4 by FOXP4-AS1 *via* sequestering miR-3184-5p, and downregulation of the latter in PCa tissues predicts poor OS (14). Zhong et al. showed that FOXP4-AS1 upregulated *STMN1* through interaction with miR-423-5p as a competitive endogenous RNA, thereby regulating NPC progression (16). FOXP4-AS1 inhibition up-regulated *CBX4 via* miR-136-5p to suppress migration, proliferation and invasion of CC cells (11). FOXP4-AS1 was overexpressed in CRC and promoted cancer cell survival and proliferation by downregulating *SLC25A26* (29). Furthermore, FOXP4-AS1 could also positively regulate their expression levels though binding to *LSD1* and *EZH2* to develop a carcinogenic complex, hence expediting GC cells to invade, proliferate and migrate (21). Moreover, FOXP4-AS1 and its functional roles and related genes were determined to further explore the relationship between FOXP4-AS1 and various cancers (**Table 4**).

**Table 4:** Summary of FOXP4-AS1 functional roles and related genes.

Cancer	Expression	Functional role	Target genes	Experiment Type	Reference
CRC	Upregulate	Cell proliferation and apoptosis	SMIM4, SAYSD1, SLC25A26	*In vitro* and *in vivo*	(12, 27)
ESCC	Upregulate	Cell proliferation and apoptosis	miR-3184-5p, IGF2BP2	*In vitro*	(30)
GC	Upregulate	Cell proliferation, migration, and invasion	EZH2, LSD1	*In vitro*	(21)
NPC	Upregulate	Cell proliferation and apoptosis	miR-423-5p, STMN1	*In vitro* and *in vivo*	(16)
CC	Upregulate	Cell proliferation, migration, and invasion	miR-136-5p, CBX4	*In vitro*	(11)
OSA	Upregulate	Cell proliferation, migration, invasion, and cell cycle	LATS1, LSD1, EZH2	*In vitro*	(13)
PCa	Upregulate	Cell proliferation and apoptosis	miR-3184-5p, PAX5	*In vitro* and *in vivo*	(14)
MCL	Upregulate	Cell proliferation, migration, and invasion	miR−423−5p, NACC1	*In vitro*	(22)

CRC, colorectal cancer; ESCC, esophageal squamous cell carcinoma; GC, gastric cancer; NPC, nasopharyngeal carcinoma; CC, cervical cancer; OSA, osteosarcoma; PCa, prostate cancer; MCL, mantle cell lymphoma.

## Discussion

With the advancement of high-throughput sequencing technology, lncRNA-related studies are gradually increasing. Increasing evidence demonstrates that lncRNAs were aberrantly expressed in a variety of malignancies, act as oncogenes or tumor suppressors based on their expression levels, and play a role in cancer development. For example, lncRNAs, such as PANDAR (31), MIAT (32), and SNHG15 (33) have been shown to be aberrantly expressed in various tumors and interrelated with the prognosis of tumor patients. Together, these findings suggested that lncRNA could be a distinct cancer prognostic biomarker and therapeutic target.

LncRNA FOXP4-AS1 levels are consistently higher in tumor tissues compared to normal tissues, and the overexpression of FOXP4-AS1 is interrelated with the progression and prognosis of various cancers (12, 16–18). Studies have increasingly shown that upregulation of FOXP4-AS1 accelerates tumor development and progression by promoting tumor cell metastasis, proliferation, and inhibiting apoptosis (22, 30). Nevertheless, the prognostic impact of FOXP4-AS1 in pan-cancer has not been fully elucidated. Therefore, to determine the correlation of FOXP4-AS1 expression with cancers and to draw conclusions, an extensive and comprehensive study was performed.

In this meta-analysis, it was shown that FOXP4-AS1 overexpression significantly correlated with worse OS of cancer patients. Because of the obvious heterogeneity among these studies, sensitivity analysis was performed, which showed that the study by Binang et al. (20) was the main source of heterogeneity. Subgroup analysis further showed that the correlation of FOXP4-AS1 expression with OS was altered by cancer type and significantly reduced the heterogeneity, thereby indicating that heterogeneity likely originates from the different cancer types. However, meta-regression was not successful in determining the source of the obvious heterogeneity. Our data also showed that FOXP4-AS1 overexpression was positively interrelated with shorter DFS. Furthermore, it was found that FOXP4-AS1 overexpression was closely interrelated with shorter OS in HCC and NPC, whereas FOXP4-AS1 expression was not significantly interrelated with OS in GC. Therefore, the prognostic value of FOXP4-AS1 is possibly different across cancer types. Moreover, the relation of FOXP4-AS1 expression with clinicopathological features was studied, and the results indicated that FOXP4-AS1 overexpression was also remarkably interrelated with a larger tumor size. Overall, our data demonstrated that FOXP4-AS1 could be a potential and noninvasive prognostic biomarker in various human cancers.

The target genes of FOXP4-AS1 were further predicted and functionally annotated. *SNORA56*, *SRPK1*, and *PRICKLE4* were significantly co-expressed with FOXP4-AS1 and played significant roles in breast cancer (34), NPC (35), HCC (36), lung adenocarcinoma (37), colon cancer (38) and ovarian cancer (39). Additionally, it has been shown that FOXP4-AS1 was interrelated with various functions, including double-strand break repair, DNA repair, and post-translational protein modification in Ewing sarcoma (40). Li et al. found through experimental studies that knockdown of FOXP4-AS1 gene affected the ratio of the number of G0/G1 and S-phase CRC cells (12). Liu et al. revealed that FOXP4-AS1 may be implicated in PDAC development by participating in biological processes such as transcription, tricarboxylic acid cycle, and oxidative phosphorylation (19). Our findings also revealed that FOXP4-AS1 may be associated with DNA replication, transcription, and cell cycle phase of tumor cells.

There are several limitations to this study that ought to be considered. First, the number of included studies was insufficient, and most had a relatively small sample size. Second, all enrolled patients were from China, which limits the generalizability of our results. Third, the biological functions of FOXP4-AS1 are different in various cancers, which increased heterogeneity. In addition, the accuracy and reproducibility of the results might be affected by extracting the survival data from the K-M curves in some studies.

In conclusion, overexpression of FOXP4-AS1 was significantly correlated with the poor OS and DFS of tumor patients. FOXP4-AS1 is a promising prognostic biomarker and potential therapeutic target in human cancer. However, further studies with a larger sample size are needed to validate the prognostic significance of FOXP4-AS1 in cancer.

## Data Availability Statement

The original contributions presented in the study are included in the article/supplementary material. Further inquiries can be directed to the corresponding authors.

## Author Contributions

GZ conceived the study; XH and HJ conducted the literature search; LF and GZ extracted the required data; TL performed the statistical analyses; GZ and YW wrote a draft; KY and HC reviewed the paper. All authors contributed to the article and approved the submitted version.

## Funding

This study was supported by The 2021 Central-Guided Local Science and Technology Development Fund (ZYYDDFFZZJ-1); Natural Science Foundation of Gansu Province, China (No. 18JR3RA052); Lanzhou Talent Innovation and Entrepreneurship Project Task Contract (No. 2016-RC-56); National Key Research and Development Program (No. 2018YFC1311506); Fundamental Research Funds for the Central Universities (No. 2020jbkyzx001; lzujbky-2020-kb20); Guiding plan for scientific and technological development of Lanzhou (No. 2019-ZD-102).

## Conflict of Interest

The authors declare that the research was conducted in the absence of any commercial or financial relationships that could be construed as a potential conflict of interest.

## Publisher’s Note

All claims expressed in this article are solely those of the authors and do not necessarily represent those of their affiliated organizations, or those of the publisher, the editors and the reviewers. Any product that may be evaluated in this article, or claim that may be made by its manufacturer, is not guaranteed or endorsed by the publisher.
